# From general practice in Australia to academic family medicine in Malaysia

**DOI:** 10.51866/mol.888

**Published:** 2025-05-04

**Authors:** Mohamed-Syarif Mohamed-Yassin

**Affiliations:** 1 MBBS, FRACGP, DipPallMed(Clin.), Department of Primary Care Medicine, Universiti Teknologi MARA, Sungai Buloh Campus, Sungai Buloh, Selangor, Malaysia. Email: syarif8258@uitm.edu.my

**Keywords:** General practitioner, Family physician, Academia, Family medicine specialist, Primary care

Over the years, I often get asked why I returned to Malaysia after working as a general practitioner in Australia. I first arrived in Australia in 2001, aged 19, to study medicine at Monash University, Victoria. Three years after graduation, I began my general practice training in two rural settings in South Australia. After obtaining fellowship from the Royal Australian College of General Practitioners, I joined a practice in Adelaide’s northeastern suburbs. My wife, whom I met at Monash, and I spent almost 13 years studying and working in Australia. Our two children were also born there.

We contemplated returning to Malaysia on many occasions. As our eldest child approached primary school age, we decided it was time to return ‘home’ to reconnect with our extended family who remained there. We wanted our children to be closer to their grandparents as well as their uncles, aunts and cousins.

Once we were back, my parents stayed with us for short periods - *Popo* (grandmother in Mandarin; she is Chinese) helped care for our children, while *Grandpa* drove them to and from school. We went on several road trips together (Ipoh, Penang, Malacca and Johore), and our children grew close to their *Popo, Grandpa* and *Auntie Syamsiah.*

We also spent a lot of time with our children’s *Tokwan* and *Maktok* (their grandparents in Kedah), where our children developed strong bonds with their cousins. Together, we explored the states of Perak, Pahang, Terengganu, Sarawak and Sabah. These trips exposed our children to diverse local cuisines and cultural traditions. They became more fluent in Malay, and they also picked up a bit of Mandarin.

There were challenges with this move from Australia to Malaysia. Professionally, I decided to join the medical faculty of a public university in Selangor, transitioning from a full-time general practitioner to an academic family physician. This change resulted in a significant drop in income - I earned about one-sixth of the amount in Adelaide.

Another challenge was adapting to academia, especially the research component. I had to learn about research methodology, biostatistics, and postgraduate supervision, and improve my academic writing. Lifelong learning, a principle often emphasised during medical training, was truly put to the test. Along with courses, several books were helpful: *A Step by Step Guide to Primary Care Research* by the Malaysian Primary Care Research Group, *Helping Doctoral Students Write: Pedagogies for Supervision* by Kamler and Thomson and *Membina Kerjaya Akademia* by Mohd Azraai Kassim.

I also had to brush up on common Malaysian medical conditions, such as dengue fever and tuberculosis, which are uncommon in Australia. Chronic diseases such as diabetes mellitus, hypertension, dyslipidaemia and chronic kidney disease are also more prevalent here, and often present at advanced stages. Still, I truly enjoy being able to converse with my patients in various languages, including Malay, English, Mandarin and even Manglish (Malaysian English)!

Reflecting on the 9 years since we returned, I have no regrets. This decision afforded us meaningful time with family before my father’s passing 2 years ago.^1^ My children still recall how Grandpa would stop at a nearby convenience store after school and buy them sweet treats. Memories like this remind me why returning to Malaysia was the right choice.
